# Gender and respiratory findings in workers occupationally exposed to organic aerosols: A meta analysis of 12 cross-sectional studies

**DOI:** 10.1186/1476-069X-8-1

**Published:** 2009-01-12

**Authors:** E Neil Schachter, Eugenija Zuskin, Erin L Moshier, James Godbold, Jadranka Mustajbegovic, Jasna Pucarin-Cvetkovic, Angelo Chiarelli

**Affiliations:** 1 Mount Sinai School of Medicine, 1 Gustave L. Levy Place 1232, New York, NY, USA; 2 Andrija Stampar School of Public Health, Rockefellerova 4 Zagreb, Croatia

## Abstract

**Background:**

Gender related differences in respiratory disease have been documented. The aim of this study was to investigate gender related differences in respiratory findings by occupation. We analyzed data from 12 of our previously published studies.

**Methods:**

Three thousand and eleven (3011) workers employed in "organic dust" industries (1379 female and 1632 male) were studied. A control group of 806 workers not exposed to any kind of dust were also investigated (male = 419, female = 387). Acute and chronic respiratory symptoms and lung function were measured. The weighted average method and the Mantel-Haentszel method were used to calculate the odds ratios of symptoms. Hedge's unbiased estimations were used to measure lung function differences between men and women.

**Results:**

There were high prevalences of acute and chronic respiratory symptoms in all the "dusty" studied groups compared to controls. Significantly less chronic cough, chronic phlegm as well as chronic bronchitis were found among women than among men after the adjustments for smoking, age and duration of employment. Upper respiratory tract symptoms by contrast were more frequent in women than in men in these groups. Significant gender related lung function differences occurred in the textile industry but not in the food processing industry or among farmers.

**Conclusion:**

The results of this study suggest that in industries processing organic compounds there are gender differences in respiratory symptoms and lung function in exposed workers. Whether these findings represent true physiologic gender differences, gender specific workplace exposures or other undefined gender variables not defined in this study cannot be determined. These data do not suggest that special limitations for women are warranted for respiratory health reasons in these industries, but the issue of upper respiratory irritation and disease warrants further study.

## Background

Our studies as well as those of others demonstrate the adverse respiratory effects of exposure to organic dusts in the workplace. We have studied workers in the textile industry (cotton, flax, wool, jute, sisal and hemp) food processing industry (i.e. green and roasted coffee, tea, spices, dried fruits, cocoa, flour, soy) as well as in farming [1-12]. The current analysis is based on data from12 previously published studies.

There are now several studies examining respiratory function differences between women and men which suggest that women may represent a more vulnerable population for respiratory disease. However, most of these studies deal with the general population. For example, community based epidemiologic studies have shown that women with cough experience an accelerated loss in FEV1 compared to men with similar symptoms [13]. The data of Chen et al. [14] suggests that cigarette smoking may be more detrimental in its effects on lung function in women than among men. This group [14] also documented that FEV1 and MMFR decreased with increasing pack-years more rapidly in women than in men. Similarly, Xu et al. [15,16] reported that the effects of smoking on pulmonary function were greater in women than in men. By contrast, Walter and Richard [17] in a study of Indian men and women showed that the decline in FEV1 and FEF25-75 was significantly less in women than in men. By studying a general population, Jaen et al.[18] found a higher prevalence of chronic bronchitis in men (21%) than in women (2.7%) as well as a higher prevalence of dyspnea (men: 11.4%; women: 9.8%). The prevalence of asthma however, was higher in women (4.4%) than in men (2.1%). In their study the prevalence of clinically significant airflow limitation was found in 10.4% of men compared to 4.1% of women. Enright et al. [19] reported asthma in 8.6% of women and in 9.4% of men with greater peak flow lability in women (12%) than in men (10%). By contrast, Urlik and Lange [20] found a similar prevalence of asthma in men (3.7%) and in women (3.6%). Moreover, FEV1, FVC and FEV1/FVC% as a percentage of predicted are significant risk factors for mortality in both men and women [21,22].

Relatively few studies compare the effect of occupation on respiratory function by gender. Lemasters et al. [23] studied the respiratory health of workers employed in the manufacturing of ceramics. They indicated that there may be important sex differences in the response to occupational and/or environmental exposures between men and women. In their study there were larger FVC decreases in women compared to men. Additionally, in a study of Piitulainen et al. [24] lung function was lower in nonsmoking men than in nonsmoking women occupationally exposed to airway irritants (gases, fumes or dust). The same authors suggested that men are at greater risk of lung function deterioration in this setting than women.

Mustajbegovic et al. [25] reported that in chemical workers there were higher prevalences of chronic respiratory symptoms among women than men workers. Spirometric abnormalities among workers in hard metal plants were more frequent in women than in men. Jarvis et al. [26] reported that women may be more susceptible than men to the products of gas combustion. Viegi et al. [27] however, found no significant difference for symptom prevalence rates between men and women exposed to gas, chemicals or dusts.

The present investigation was undertaken to study possible gender related differences due to occupational exposure to organic dusts in workers studied in 12 previously reported epidemiological studies. These studies used a standardized questionnaire and the same lung function equipment and methodology for evaluation of workers.

### Subjects and methods

Over the past 20 years we have investigated industries associated with organic dust. In all cases the industrial physicians associated with each industry was contacted and these medical personnel put us in contact with management. After explaining the purpose of our study to management we set up a standardized testing methodology for studying the respiratory health of the workforce. Workers were invited to participate in a meeting just before the work shift so that we could explain to them the purpose and the methods of the study. While some industries which were contacted failed to cooperate the vast majority were compliant and a majority of available workers participated in the studies.

The workers in this study were exposed to a variety of organic dusts. Details of the work environment of these industries and the nature of the contaminations to which the the workers were exposed is found in references 1–12. Endotoxin measurements were not performed in these industries. We studied a total of 3011 workers (1379 female and 1632 male). Workers in the food processing industries (N = 746 female and N = 259 male) were exposed to aerosols and dusts of coffee, tea, spices, soy, animal feed, dried fruits, cocoa and flour. A group of 381 female and 173 male textile workers were exposed to cotton, hemp, wool, jute and sisal. Farm workers (252 female and 1,200 male) were exposed to grain as well as livestock in swine confinement buildings and poultry coops (see Table 1).

**Table 1:** Chronic respiratory symptoms in exposed female and male workers in food processing, textile and farming industries

Group	Sex	N	Mean age (yrs)	Mean exposure (yrs)	Smoking habit pack year	Chronic cough %	Chronic phlegm %	Chronic bronchitis %	Occupational asthma %	Dyspnea grade 3&4 %	Nasal catarrh %	Sinusitis %
All food processing workers	F	746	33	10	5.0	24.9	19.1	14.9	2.4	21.4	16.3	19.8
	M	259	35	10	14.4	39.3	36.4	29.4	1.8	25.4	30.1	18.6

All textile workers	F	381	31	9	6.0	27.2	16.8	14.3	4.9	20.8	27.3	16.5
	M	173	35	11	13.8	43.1	32.8	29.9	4.2	20.4	18.5	13.9

All farmers	F	252	39	11	8.4	21.6	14.3	9.6	2.3	24.4	21.1	17.1
	M	1200	36	11	16.8	31.8	27.6	24.4	1.6	13.3	18.6	18.0

F = female

M = male

The mean age of the female workers was 34 years (range 23 to 44 years) and for men it was 35 years (range: 25 to 43 years). The mean duration of exposure to organic dusts for female workers was 10 years (range: 3 to 13 years) and for male workers it was 11 years (range: 4 to 18 years). Differences in smoking were notable. Women were primarily nonsmokers (NS) (NS = 65%) while men were predominantly smokers (S) (S = 88%). Men had smoked on average 13 pack years and women on average 7 pack years.

After obtaining the co-operation of company management and health authorities in each industry we worked with these individuals to obtain the approval and consent of the studied individuals. In general, we were able to study over 80% of the workforce in each of the industries examined. Informed consent approved by the human investigation committee of the University of Zagreb was obtained for each of the studied workers. These studies were performed between 1985 and 2006. None of the industries which we approached refused to participate in these studies.

### Respiratory symptoms

Chronic respiratory symptoms were recorded by using the British Medical Research Council questionnaire on respiratory symptoms [28] with additional questions for occupational asthma [29-31]. For all workers, a detailed occupational history as well as questions about smoking habits were recorded. The following definitions were used:

Chronic cough or phlegm: cough and/or phlegm for a minimum of three months a year for at least one year;

Chronic bronchitis: cough and phlegm for a minimum of three months a year and for not less than 2 successive years;

Dyspnea grades: 3 – shortness of breath when walking with other people at an ordinary pace on level ground; grade 4 – shortness of breath when walking at their own pace on level ground;

Occupational asthma: a diagnosis confirmed by the medical officer of the plant and based on records characterizing reversible airway obstruction historically related to the work place.

The data on nasal catarrh and sinusitis were obtained from the medical records in the industrial health center.

Acute symptoms that developed during the work shift were also recorded in all studied workers. These symptoms included dry cough; dyspnea; irritation or dryness of the throat; secretions; dryness or bleeding from the nose; eye irritation; headache.

All industries including the farm workers were serviced by medical doctors trained in occupational medicine at the Medical School in Zagreb. They used standardized definitions of asthma.

### Lung function measurements

In all the studied industries, ventilatory capacity measurements were performed by recording maximum expiratory flow-volume (MEFV) curves on a spirometer, the Pneumoscreen (Jaeger, Wurzburg, Germany). The forced vital capacity (FVC), the one-second forced expiratory volume (FEV1), and maximum flow rates at 50% and the last 25% of the vital capacity (FEF50, FEF25) were read on these MEFV curves. In food processing workers and in textile workers measurements were performed in the morning before the work shift (6 am) and again after the work shift (2 pm). In farm workers, the testing was performed only once during the morning work shift. Spirometers were calibrated on a daily basis. Lung function testing was performed according to the recommendations of Quanjer et al. [32]. At least three MEFV curves were recorded for each subject and the best value of the three technically satisfactory MEFV curves (the best FVC and FEV1) was used as the result of the test. The measured values of ventilatory capacity were compared with the predicted normal values of Quanjer [33].

### Environmental measurements

The dust concentrations in the work environment were measured by a two stage Hexhlet apparatus (Casella, London, England) which collects total and respirable dust particles. Nine to ten dust samples were collected during over an 8-hour work shift in areas where subjects were working. Arithmetic means and ranges were measured as mg/m^3^. These findings have been reported in our previous studies on individual industries [1-9].

### Statistical analysis

The chi-square test (or when appropriate Fisher's exact test), was used for testing differences in the prevalence of respiratory symptoms between groups. Odds ratios for the presence of respiratory symptoms assessing the relative odds of developing individual symptoms among women versus men adjusted for age, years of exposure, amount and frequency of smoking were calculated for each industry and respiratory symptom [34]. Adjustment for age, years of exposure, amount and frequency of smoking were made only when the cell frequencies were non-zero. The Mantel-Haenszel method was used when the industry had a small or zero cell frequency. When the cell frequencies were neither small nor 0, the weighted average (weighted by the inverse of the variance) method proposed by Woolf [35] was used to calculate a combined estimate of the odds ratio. Both methods were based on the assumption that the odds ratios were constant across industries. Hence a test for homogeneity of odds ratios across industries for each symptom was performed prior to calculating the common odds ratio. If the estimates were inhomogeneous, a common estimate was not considered as valid.

The results of ventilatory capacity measurements were analyzed by the paired t-test when comparing baseline to predicted values. A level of p < 0.05 was considered statistically significant. Estimates of mean differences in lung function and across shift changes in lung function between women and men in each industry were obtained using Hedges' estimator, an unbiased estimator that corrects for small sample sizes [36].

To obtain common or combined mean differences across industries, a weighted average of the mean differences was obtained. Each difference was weighted by the inverse of its variance to obtain a pooled estimate of the combined estimator for the mean difference. In order for this combined estimate to be valid, the effect-size estimates must be homogeneous across industries. A test for homogeneity was performed prior to combining the industry estimates.

## Results

### Respiratory symptoms

#### Chronic symptoms

Table 1 shows the prevalence of chronic respiratory symptoms by gender for food processing workers, textile workers and farmers. The prevalence of chronic respiratory symptoms by specific industry is presented in Tables 2 for food processing workers in Table 3, for textile workers and in Table 4 for farmers. The highest symptom prevalences across all exposed groups were recorded for chronic cough (female: 6.0% to 50.0%; male: 24.8% to 60.0%), followed by chronic phlegm (female: 6.0% to 28.9%; male: 22.8% to 61.9%), dyspnea (female: 9.2% to 57.6%; male: 5.5% to 33.3%) and nasal catarrh (female: 5.7% to 65.9%; male: 4.1% to 38.7%). Female workers complained of significantly less chronic phlegm than did men (p < 0.01) but had significantly higher prevalences of nasal catarrh and dyspnea than men (p < 0.01). The highest prevalence of occupational asthma in women was found in agricultural workers (10.5%) and the highest prevalence in men was found in workers processing soy beans (7.4%). (For details of chronic symptoms by individual industries see tables 2, 3, 4)

**Table 2:** Chronic respiratory symptoms in exposed female and male workers in food processing industries

Group	Sex	N	Mean age (yrs)	Mean exposure (yrs)	Smoking habit pack year	Chronic cough %	Chronic phlegm %	Chronic bronchitis %	Occupational asthma %	Dyspnea grade 3&4 %	Nasal catarrh %	Sinusitis %
Coffee	F	82	31	6	5.0	29.3	24.4	17.1	3.7	29.3	65.9	24.4
	M	21	36	9	15.0	57.1	61.9	52.4	0	33.3	38.7	23.8

Tea	F	100	34	10	5.0	29.0	15.0	13.0	4.0	26.0	36.0	15.0
	M	32	35	9	20.0	35.1	31.5	20.5	2.7	30.0	34.0	18.0

Spices	F	92	36	12	5.0	22.8	19.6	19.6	0	57.6	37.0	27.2
	M	20	35	13	15.0	31.5	30.5	24.5	0	50.2	30.0	20.1

Soy	F	31	28	8	2.5	29.7	21.8	18.5	1.5	15.2	21.7	18.2
	M	29	32	4	10.0	37.0	33.3	25.9	7.4	11.1	22.2	14.8

Animal food	F	35	38	12	7.5	20.1	28.2	20.1	1.7	9.3	20.0	16.5
	M	71	40	15	20.0	56.3	50.7	40.9	2.8	11.3	35.2	21.1

Dried fruits	F	54	30	7	5.0	16.7	12.9	12.9	0	33.3	40.7	14.8
	M	29	32	8	10.0	28.7	24.5	20.3	0	24.2	36.5	11.5

Confectionary	F	259	33	11	5.0	23.9	9.3	7.3	2.7	12.7	20.8	23.6
	M	29	31	10	10.0	27.5	20.7	20.7	0	27.6	24.1	24.1

Cocoa & flour	F	93	33	13	5.0	27.9	21.3	10.7	5.4	13.8	27.3	18.5
	M	28	35	14	15.0	41.2	38.5	30.2	1.5	15.2	20.4	15.2

All food processing workers	F	746	33	10	5.0	24.9	19.1	14.9	2.4	21.4	16.3	19.8
	M	259	35	10	14.4	39.3	36.4	29.4	1.8	25.4	30.1	18.6

F = female

M = male

**Table 3:** Chronic respiratory symptoms in exposed female and male textile workers

Group	Sex	N	Mean age (yrs)	Mean exposure (yrs)	Smoking habit pack year	Chronic cough %	Chronic phlegm %	Chronic bronchitis %	Occupational asthma %	Dyspnea grade 3&4 %	Nasal catarrh %	Sinusitis %
Cotton	F	37	34	11	7.5	31.6	15.8	15.8	3.5	31.6	52.6	37.2
	M	34	39	15	15.0	38.2	29.4	23.5	6.5	23.5	32.9	20.3

Hemp	F	48	35	12	7.5	50.0	28.9	26.3	10.5	21.1	39.5	21.1
	M	29	43	18	23.0	57.1	39.3	39.3	7.1	21.4	25.0	25.0

Wool	F	176	35	11	7.5	24.5	11.0	6.0	3.5	18.5	28.5	20.4
	M	76	36	11	16.0	60.8	44.8	43.5	4.2	25.8	21.7	22.5

Jute	F	70	23	3	2.5	15.7	14.3	11.4	2.9	22.9	5.7	0
	M	15	25	4	5.0	30.5	25.1	19.1	1.5	18.7	4.1	0

Sisal	F	50	29	8	5.0	14.0	14.0	12.0	4.0	10.0	10.0	4.0
	M	19	30	9	10.0	29.1	25.5	24.1	1.9	12.4	9.0	1.5

All textile workers	F	381	31	9	6.0	27.2	16.8	14.3	4.9	20.8	27.3	16.5
	M	173	35	11	13.8	43.1	32.8	29.9	4.2	20.4	18.5	13.9

F = female

M = male

**Table 4:** Chronic respiratory symptoms in exposed female and male farmers

Group	Sex	N	Mean age (yrs)	Mean exposure (yrs)	Smoking habit pack year	Chronic cough %	Chronic phlegm %	Chronic bronchitis %	Occupational asthma %	Dyspnea grade 3&4 %	Nasal catarrh %	Sinusitis %
Swine	F	18	38	10	7.5	50.0	27.8	16.7	0	50.0	22.2	19.7
	M	41	32	8	13.0	41.5	36.6	31.7	0	21.9	21.9	20.5

Poultry	F	91	37	12	7.5	19.8	14.3	12.1	1.1	9.9	23.1	20.5
	M	252	37	9	17.0	33.7	27.4	24.1	1.2	5.5	17.7	22.6

Agriculture	F	76	44	10	10.0	10.5	9.2	6.6	6.6	9.2	21.1	18.5
	M	738	38	15	20.0	24.8	22.8	20.1	1.5	9.1	20.7	19.5

Live- stock	F	67	36	11	8.5	6.0	6.0	3.0	1.5	28.3	17.9	9.7
	M	169	35	11	17.0	27.2	23.7	21.9	3.6	16.8	14.2	9.5

All farmers	F	252	39	11	8.4	21.6	14.3	9.6	2.3	24.4	21.1	17.1
	M	1200	36	11	16.8	31.8	27.6	24.4	1.6	13.3	18.6	18.0

F = female

M = male

The odds ratios and confidence intervals for developing chronic symptoms in women versus men are detailed in Table 5, which analyze the food processing, textile and agricultural industries respectively. Examination of the data indicates that women were less likely than men to develop chronic cough, phlegm or chronic bronchitis in all three industries. Women agricultural workers were more likely than men to develop occupational asthma.

**Table 5:** Odds ratios of women v. men for developing chronic symptoms

	Food Processing		Textile Industry		Farming Industry	
**Chronic Symptoms**	**Odds Ratio**	**Confidence Interval**	**Odds Ratio**	**Confidence Interval**	**Odds Ratio**	**Confidence Interval**

Chronic Cough	0.50	0.36 – 0.69+	0.35	0.23 – 0.52+	0.39	0.26 – 0.57+

Chronic Phlegm	0.31	0.22 – 0.44+	0.32	0.21 – 0.49+	0.38	0.25 – 0.58+

Chronic Bronchitis	0.33	0.22 – 0.49+	0.27	0.17 – 0.43+	0.30	0.18 – 0.49+

Occupational Asthma	1.69	0.53 – 2.86	1.05	0.41 – 2.65	1.72	0.86–2.58

Dyspnea 3 & 4	1.07	0.72 – 1.58	0.80	0.51 – 1.26	*	*

Sinusitis	1.18	0.79 – 1.75	1.13	0.64 – 1.62	1.03	0.70 – 1.51

Nasal Catarrh	1.04	0.74 – 1.47	1.41	0.89 – 2.25	1.30	0.92 – 1.84

* Not homogeneous, cannot combine odds ratios across industries.

+ Significant odds ratios.

#### Acute symptoms

The prevalence of acute symptoms in exposed workers is presented in Table 6 for food processing workers, for textile workers and for farmers. The prevalence of acute respiratory symptoms by specific industry is presented in Tables 7 for food processing workers in Table 8, for textile workers and in Table 9 for farmers. High prevalences were found particularly for cough (female: 33.3% to 76.3%; male: 30.5% to 71.4%), for dyspnea (female: 33.3% to 65.8%; male: 26.0% to 71.4%), for irritation and dryness of the throat (female: 15.3% to 77.8%; male: 16.2% to 67.9%) for eye irritation (female: 28.9% to 77.8%; male: 29.4% to 82.1%) and dryness of the nose (female: 20.0% to 65.8%; male: 15.1% to 61.9%). Similar prevalences of acute symptoms were found in both male and female workers in all studied groups. (For details of acute symptoms by individual industries see Tables 7, 8, 9).

**Table 6:** Acute symptoms in exposed female and male workers in food processing, textile and farming industries

Group	Sex	N	Cough %	Dyspnea %	Throat		Eye irritation %	Nose			Headache %
								
					irritation %	dryness %		secretion %	dryness %	bleeding %	
All food processing workers	F	746	45.8	39.7	42.7	36.2	41.4	21.0	41.5	24.5	32.3
	M	259	39.7	44.5	42.7	37.7	42.0	24.3	39.3	19.2	28.0

All textile workers	F	381	65.6	55.8	59.3	59.9	62.8	26.3	38.7	25.0	44.4
	M	173	64.1	56.6	56.2	54.3	58.7	24.0	37.9	24.3	31.5

All farmers	F	252	53.3	44.9	48.8	35.9	44.7	19.7	31.7	10.9	28.4
	M	1200	48.2	41.0	34.7	33.7	36.9	17.1	29.4	17.2	13.0

F = female

M = male

**Table 7:** Acute symptoms in exposed female and male workers in food processing industries

Group	Sex	N	Cough %	Dyspnea %	Throat		Eye irritation %	Nose			Headache %
								
					irritation %	dryness %		secretion %	dryness %	bleeding %	
Coffee	F	82	37.8	43.9	45.1	34.1	63.4	29.3	36.6	31.7	39.0
	M	21	57.1	52.4	61.9	42.9	47.6	52.4	61.9	33.3	47.6

Tea	F	100	58.7	42.4	56.5	27.2	30.4	25.0	47.8	40.2	28.3
	M	32	50.6	40.2	50.5	20.3	29.5	15.0	39.8	20.1	18.4

Spices	F	92	58.7	42.4	56.5	27.2	30.4	25.0	47.8	40.2	28.3
	M	20	50.1	45.2	57.6	20.2	29.4	20.1	40.5	30.2	20.4

Soy	F	31	48.5	40.4	39.5	20.7	28.9	24.2	39.1	10.5	11.4
	M	29	55.6	48.2	44.4	40.7	44.4	33.3	40.7	13.8	25.9

Animal Food	F	35	50.1	39.5	36.5	41.2	38.9	10.5	41.5	10.2	36.8
	M	71	53.5	47.9	46.5	54.9	53.5	8.5	53.5	9.9	32.4

Dried fruits	F	54	33.3	33.3	25.9	29.6	35.7	12.9	25.9	25.9	38.9
	M	29	30.5	33.7	20.1	25.2	30.5	10.7	20.8	20.8	18.9

Confectionary	F	259	25.5	41.0	38.5	60.3	51.3	14.1	47.4	20.5	46.2
	M	29	44.8	51.7	20.7	58.6	52.6	34.5	17.2	10.3	41.4

Cocoa & flour	F	93	54.2	34.5	43.0	49.2	52.4	27.1	46.1	17.2	29.2
	M	28	55.1	36.4	40.0	39.1	48.1	20.2	40.2	15.1	19.3

All food processing workers	F	746	45.8	39.7	42.7	36.2	41.4	21.0	41.5	24.5	32.3
	M	259	39.7	44.5	42.7	37.7	42.0	24.3	39.3	19.2	28.0

F = female

M = male

**Table 8:** Acute symptoms in exposed female and male textile workers

Group	Sex	N	Cough %	Dyspnea %	Throat		Eye irritation %	Nose			Headache %
								
					irritation %	dryness %		secretion %	dryness %	bleeding %	
Cotton	F	37	61.5	54.9	72.5	69.3	70.5	20.5	40.5	20.3	45.7
	M	34	63.7	56.9	67.3	60.5	61.3	19.5	50.0	21.4	21.5

Hemp	F	48	76.3	65.8	71.1	81.5	68.4	23.7	65.8	28.9	47.4
	M	29	71.4	71.4	67.9	67.9	82.1	17.9	60.7	28.0	82.1

Wool	F	176	67.2	60.3	50.1	40.5	41.5	30.6	30.2	29.5	35.1
	M	76	68.3	62.4	55.1	41.4	44.5	32.5	32.5	30.1	22.5

Jute	F	70	63.2	63.2	57.9	63.2	73.7	36.8	36.8	26.3	73.7
	M	15	60.1	62.3	50.7	60.3	65.5	30.7	31.2	25.1	19.3

Sisal	F	50	60.0	35.0	45.0	45.0	60.0	20.0	20.0	20.0	20.0
	M	19	57.2	30.1	40.2	41.3	40.0	19.5	15.1	17.1	12.3

All textile workers	F	381	65.6	55.8	59.3	59.9	62.8	26.3	38.7	25.0	44.4
	M	173	64.1	56.6	56.2	54.3	58.7	24.0	37.9	24.3	31.5

F = female

M = male

**Table 9:** Acute respiratory symptoms in exposed female and male farmers

Group	Sex	N	Cough %	Dyspnea %	Throat		Eye irritation %	Nose			Headache %
								
					irritation %	dryness %		secretion %	dryness %	bleeding %	
Swine	F	18	72.2	61.1	77.8	72.2	77.8	33.3	33.3	11.1	50.0
	M	41	70.7	56.1	46.3	65.9	46.3	14.6	29.3	7.3	9.8

Poultry	F	91	40.2	39.5	30.2	20.5	30.2	20.5	31.2	15.2	20.3
	M	252	39.5	37.2	30.1	24.5	35.7	27.5	30.2	17.3	15.7

Agriculture	F	76	48.5	34.5	35.0	35.5	39.5	14.5	29.5	8.1	31.0
	M	738	30.5	26.0	24.0	28.1	33.4	9.0	22.2	34.1	17.0

Livestock	F	67	52.2	44.8	52.2	15.3	31.3	10.5	32.8	9.2	12.3
	M	169	52.1	44.9	38.5	16.2	32.5	17.3	36.1	10.3	9.5

All farmers	F	252	53.3	44.9	48.8	35.9	44.7	19.7	31.7	10.9	28.4
	M	1200	48.2	41.0	34.7	33.7	36.9	17.1	29.4	17.2	13.0

F = female

M = male

The odds ratios and confidence intervals for developing acute symptoms in women versus men are detailed in Table 10. Again these refer to the food processing, textile and agricultural industries respectively. Examination of these data indicate that in the food processing industry women were less likely than men to develop cough while they were more likely to experience nose bleeds and headaches. In the textile industry, women developed more dry throat but otherwise their symptoms were similar to those of men. Because of the inhomogeneity of the odd ratios among the different plants, conclusions about the odds ratios could not be drawn for eye irritation or headache. For farm workers throat irritation and headache were more common in women; however, conclusions could not be drawn for cough, throat dryness or epistaxis.

**Table 10:** Odds ratios of women v. men for developing acute symptoms

	Food Processing		Textile Industry		Farming Industry	
**Acute Symptoms**	**Odds Ratio**	**Confidence Interval**	**Odds Ratio**	**Confidence Interval**	**Odds Ratio**	**Confidence Interval**

Cough	0.72	0.53 – 0.99+	1.06	0.72 – 1.56	*	*

Dyspnea	0.84	0.61 – 1.14	0.92	0.63 – 1.33	1.17	0.88 – 1.56

Throat Irritation	1.17	0.85 – 1.61	1.23	0.85 – 1.79	1.49	1.12 – 1.98+

Throat Dryness	1.09	0.79 – 1.50	1.64	1.13 – 2.39	*	*

Eye Irritation	1.04	0.76 – 1.42	*	*	1.21	0.91 – 1.61

Nasal Secretions	0.79	0.54 – 1.15	0.91	0.60 – 1.37	0.83	0.56 – 1.23

Nasal Dryness	1.30	0.94 – 1.79	1.15	0.77 – 1.70	1.05	0.78 – 1.42

Nose Bleeds	1.67	1.11 – 2.52+	0.93	0.61 – 1.41	*	*

Headache	1.42	1.01 – 1.99+	*	*	1.97	1.38 – 2.80+

* Not homogeneous, cannot combine odds ratios across industries.

+ Significant odds ratios.

### Lung function measurements

Table 11 compares baseline lung function differences and across shift changes in lung function between women and men in the 3 industry groups. In the food processing industry there was no difference attributable to gender for FEF25, FEF50 and FEV1. For across shift changes in lung function there was no gender difference for FEF50. For the other lung function parameters common odds ratios could not be provided due to inhomogeneity of odds ratios among food processing industries.

**Table 11:** Standardized mean difference between women and men for lung function parameters

	Food Processing		Textile Industry		Farming Industry	
**Lung Function Parameters**	**Standardized Mean Difference**	**Confidence Interval**	**Standardized Mean Difference**	**Confidence Interval**	**Standardized Mean Difference**	**Confidence Interval**

FEF25	0.0322	(-) 0.1219 – 0.1863	0.2479	0.0643 – 0.4315+	0.0105	(-) 0.1294 – 0.1505

FEF50	0.0583	(-) 0.0959 – 0.2125	0.2171	0.0336 – 0.4005+	(-) 0.0287	(-) 0.1687 – 0.1113

FEV1	0.1524	(-) 0.0022 – 0.3070	*	*	*	*

FVC	*	*	0.2787	0.0951 – 0.4624+	*	*

A FEF25	*	*	0.2954	0.1117 – 0.4792+	NO	DATA

A FEF50	0.0015	(-) 0.1528 – 0.1557	0.1290	(-) 0.0541 – 0.3121		

A FEV1	*	*	*	*		

A FVC	*	*	*	*		

A = Across shift change

* Not homogeneous cannot combine differences across industries

+ Significant standardized  mean differences

In the textile industry significant differences were seen for FEF25, FEF50 and FVC with women having better lung function than men. For across shift differences there were significant gender effects for FEF25, with women having less across shift reduction than men. No difference was seen for FEF50. For all other lung function parameters, the inhomogeneity of odds ratios prevented the use of a common estimate of the odds ratio among textile industries.

In farm workers no significant differences were seen for baseline lung function by gender for FEF25, FEF50; FEV1 and FVC could not be analyzed because of inhomogeneities. Across shift change data were not available.

### Environmental measurements

Environmental measurements at various work sites in the studied industries demonstrated elevated dust levels. The highest concentrations of dust were seen in the textile industries (mean total dust: 25 mg/m^3^; mean respirable fraction: 11 mg/m^3^) followed by the food processing industries (mean total dust: 12 mg/m^3^, mean respirable fraction: 5 mg/m^3^) and at agricultural work sites (mean total dust: 9 mg/m^3^, mean respirable fraction: 4 mg/m^3^). In the textile industries the total dust concentrations ranged between 0.43 to 68,5 mg/m^3 ^and the respirable fraction between 0.7 to 45.6 mg/m^3^. In food processing industries the total dust concentrations ranged between 0.12 to 35.6 mg/m^3 ^and respirable fraction between 0.5 to 6.6 mg/m^3^. For agricultural workers the total dust concentrations varied between 3.0 and 21.5 mg/m^3 ^and for respirable fraction between 0.10 and 3.1 mg/m^3^.

## Discussion

The workers in the reported studies were exposed to a wide variety of organic dusts. There were high prevalences of chronic respiratory symptoms at these work sites for all workers, compared to controls [1-9] being highest for chronic cough, chronic phlegm, dyspnea and nasal catarrh. Our odds ratio analysis indicates that overall men were more likely to exhibit more respiratory symptoms (independent of smoking) than women. These findings are in concordance with earlier studies. In the study of Neukirch and Perdrizet [37] chronic bronchitis was found in 15% of men and 8% of women. Littlejohns et al. [38] also studied prevalences of chronic respiratory disease and found that chronic bronchitis affected 17% of men but only 7% of women. They also reported wheezing in 9% of men and 3% of women.

The prevalence of occupational asthma among our studied workers varied from 1.1% to 10.5% in female workers and from 1.2% to 7.4% in male workers. No significant differences in prevalence were elicited by odds ratio analysis for food processing or textile workers. Among farm workers women had a higher prevalence of occupational asthma than men. In a study of Mustajbegovic et al. [25] occupational asthma was reported in 0.5% of men and in 1.5% of the women occupationally exposed to low concentrations of organic and inorganic air pollutants in the chemical industry. Sobradillo et al. [39] diagnosed occupational asthma in 4.2% to 5.5% of women and 3.8% to 5.9% of men in the general population. Nejjari et al. [40,41] studied the prevalence of asthma related to occupation and found that the prevalence rate in men was 7.3% and in women, 5.2%. In their study occupational asthma was particularly high in farm workers (13%). The same authors reported the prevalence of chronic bronchitis in 20% of male and 8% of females [40].

In the USA it has been suggested that there may exist a gender bias in the diagnosis of asthma and COPD with women preferentially receiving the diagnosis of asthma and men that of COPD [42,43]. In Croatia we know of no literature to suggest such a bias and hence we feel it is unlikely that the excess of occupational asthma in female agricultural workers is due to a gender bias for the diagnosis of asthma among women. In general, epidemiologic surveys find an excess of asthma among adult females compared to males, hence these findings in agricultural workers may reflect an accentuation of this natural difference brought about by environmental antigens.

A large number of our workers complained of acute symptoms that developed during the work shift. The prevalence of these symptoms was similar in female and male workers, being highest for cough, dyspnea, throat and eye irritation and nasal catarrh. Our odds ratio analysis however, does suggest that in many of these industries women have more acute upper respiratory symptoms than men. By contrast Hytonen et al. [44,45] studied the risk of occupational rhinitis and found that while men seemed to develop this form of rhinitis early in their work history women caught up by the time they were in their 40's.

The workers in our studies demonstrated significant across shift reductions primarily for FEF50 and FEF25 and had significantly lower baseline tests than the predicted values. Our analysis of lung function in the studied industries failed to show gender differences, with the exception of the textile industry workers (our dustiest industry) where men experienced greater across shift differences and lung function declines than did women.

While there are few studies of gender and respiratory function in the occupational setting, a number of studies suggest that women may be more resistant to the effects of irritants. Krzyzanowski et al. [46] reported that pulmonary function is reduced for several years after a single chest cold in men but only after multiple chest colds in women. By studying lung function related to occupation, Krzyzanowski and Kauffmann [47] reported that among men, FEV1/FVC and FEF25-75%FVC was significantly lower among men with workplace exposures than among men never exposed. In the same study among women, occupational exposure was only significantly related to a lower FEV1/FVC.

A healthy worker effect, namely over-representation of workers who are more resistant to the workplace environment is always a potential shortcoming in a cross-sectional study or more generally any epidemiologic study that does not examine both current workers and workers who have left the industry, or retired. Our study did not have the opportunity to examine these important groups, so that in fact the work place effect may be even more serious than that reported here. The fact that symptoms and lung function were abnormal among those currently employed therefore does suggest a work place effect on health.

## Conclusion

The potential for developing respiratory disease in workers employed in industries which use organic materials is high. The patterns of these diseases may reflect gender differences. Our meta-analysis indicates that independently of age and smoking, lower respiratory symptom abnormalities are in general, more common in men than women, whereas some upper respiratory symptoms appear to be more common in women. The exception of occupational asthma in female agricultural workers will require further study. Lung function differences may indicate a greater overall risk of impairment for men than women particularly in the textile industries. Whether these findings represent true physiologic gender differences, gender specific workplace exposures or other undefined gender variables not defined in this study cannot be determined. These data do not suggest that special limitations for women are warranted for respiratory health reasons in these industries, but the issue of upper respiratory irritation and disease warrants further study.

## Abbreviations

(**MEFV**): Maximum expiratory flow-volume; (**FVC**): Forced vital capacity; (**FEV1**): One-second forced expiratoryvolume; (**FEF50, FEF25**): Maximum flow rates at 50% and the last 25% of the vital capacity.

## Competing interests

The authors declare that they have no competing interests.

## Authors' contributions

ENS and EZ participated in design and planning the study. EM and JG wrote the statistical program and performed the data analysis. JM participated in the field study and data collection. JPC and AC contributed in writing the manuscript and tables and revised the manuscript. All authors read and approved the final manuscript.
